# Metal- and Semimetal-Containing Inhibitors of Thioredoxin Reductase as Anticancer Agents

**DOI:** 10.3390/molecules200712732

**Published:** 2015-07-14

**Authors:** Valentina Gandin, Aristi P. Fernandes

**Affiliations:** 1 Department of Pharmaceutical and Pharmacological Sciences, University of Padova, Via Marzolo 5, 35131 Padova, Italy; 2 Division of Biochemistry, Department of Medical Biochemistry and Biophysics (MBB), Karolinska Institutet, SE-171 77 Stockholm, Sweden

**Keywords:** thioredoxin reductase, enzyme inhibitors, metal- and semimetal-based compounds, anticancer agents

## Abstract

The mammalian thioredoxin reductases (TrxRs) are a family of selenium-containing pyridine nucleotide disulfide oxidoreductases playing a central role in cellular redox homeostasis and signaling pathways. Recently, these selenoproteins have emerged as promising therapeutic targets for anticancer drug development, often being overexpressed in tumor cells and contributing to drug resistance. Herein, we summarize the current knowledge on metal- and semimetal-containing molecules capable of hampering mammalian TrxRs, with an emphasis on compounds reported in the last decade.

## 1. Introduction

The maintenance of redox homeostasis is crucial for cell survival and normal cellular function [1]. Cells regulate the redox state by balancing the generation of reactive oxygen species (ROS) with the elimination of ROS by antioxidant molecules and systems. The thioredoxin (Trx) system is central in upholding the thiol redox homeostasis within the cell and is comprised of NADPH and the redox-active thioredoxin reductase (TrxR) and Trx [2]. The thioredoxin system is involved in a wide range of biological functions within the cell, including ROS scavenging, DNA synthesis, cell proliferation, apoptosis and cell signaling [3]. TrxR is a selenoprotein, with a selenocysteine (Sec) incorporated into the active site, and belongs to a class of pyrimidine nucleotide disulfide oxidoreductases. There are three isoforms of TrxR, TrxR1 and TrxR2, which are ubiquitously expressed in cells and located in the cytosol and mitochondria, respectively, and, finally, the testis-specific TrxR3/TGR, which contains an additional glutaredoxin domain. All three isoforms are homodimeric flavoenzymes with their two subunits organized in a head to tail fashion. While the major function of TrxR is to provide electrons to Trx, having a broad substrate specificity, TrxR is also involved in a vast number of additional reactions. With the easily-accessible and highly-reactive Sec-containing active site, TrxR is capable of reducing proteins like TRP14 [4] and small molecular compounds like lipoic acid, dehydroascorbate, cytochrome c and quinones, but also hydroperoxide [5].

**Figure 1:**
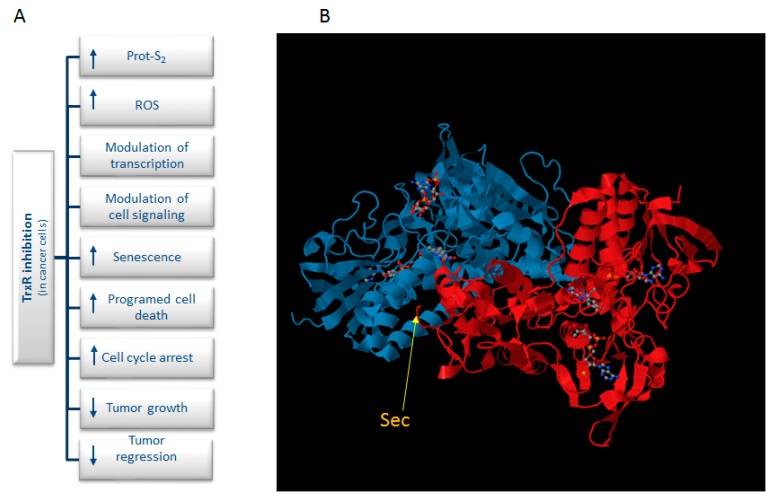
(**A**) Schematic summary of the various effects caused by TrxR inhibitors; (**B**) X-ray structure of mammalian TrxR (PDB; http://www.rcsb.org/pdb/).

Mounting evidence suggests that numerous types of cancer cells have increased levels of ROS [6]. To counteract this, cancer cells often upregulate their antioxidant defense machinery. This, however, leaves a small margin for these cells to cope with additional stress. Cancer cells with elevated ROS production are thus more vulnerable to a compromised antioxidant defense system, which will give rise to a further increase in the levels of ROS, giving rise to oxidative stress and, ultimately, cell death. Therefore, manipulating ROS levels by redox modulation is an encouraging way to selectively kill cancer cells without causing significant toxicity to normal cells [7,8]. Moreover, the expression levels of the cytosolic isoform Trx1 and TrxR1 are increased in several human carcinomas [9] and linked to tumor aggressiveness, chemo-resistance and to resistance to apoptosis [10,11,12,13,14]. In view of the above-mentioned findings, TrxR has been suggested as a promising target in cancer treatment (Figure 1A). Indeed, there are a large number of TrxR inhibitors that have been developed and identified over the years, and several of these compounds exhibit cytotoxic properties in tumor cells [15]. The TrxR inhibitors’ mode of action on TrxR inhibition is diverse and includes both competitive, noncompetitive, irreversible and mechanism-based inhibition (for more detailed examples on this matter, see [16]). The inhibitors may, for instance, act by binding to the NADPH binding site or the cysteine (Cys) or selenocysteine (Sec) residues of the two redox active sites of the enzyme (Figure 1B). Inhibitors may also bind to other sites, like the monomer to monomer interface of the homodimer. Most irreversible inhibitors of TrxR apparently act via a reaction with one or more redox-active residues (Cys and Sec), as they do not affect the enzyme in the absence of NADPH. One group of TrxR inhibitors is the metal-containing compounds, which are capable of transferring the metal ion to the catalytic Sec residue. With a highly-nucleophilic Sec residue (pKa = 5.2) positioned on its flexible and very easily-accessible carboxy-terminal position, TrxR can be selectively and irreversibly inactivated by reaction with electrophilic compounds. Conversely, such targeting of the active site of TrxR gives rise to the inhibition of the Trx system.

In recent years, a large quantity of metal-containing molecules have been found to exert their biological activities via DNA-independent mechanisms, involving enzyme inhibition pathway(s), and recent studies pointed out that TrxR constitutes an effective biomolecular target for a variety of metal-based drugs. Actually, the presence of the Sec residue in TrxR, endowed with a much higher reactivity to bind the “soft” metal ions than thiols, makes TrxR a feasible target for various metallodrugs. Herein, we provide a brief overview of metal- and semimetal-containing TrxR inhibitors, with special attention on those reported in the last decade.

## 2. Gold-Based Inhibitors

**Figure 2:**
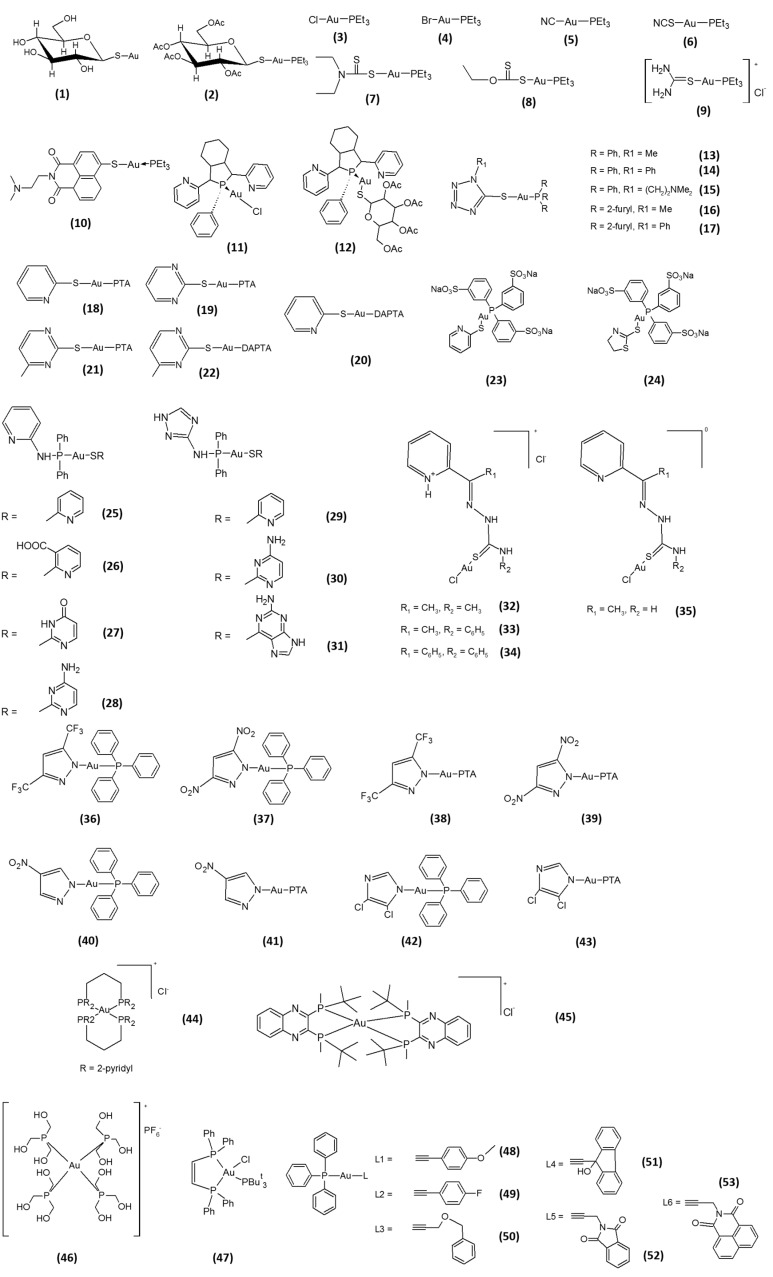
Structures of phosphine gold(I)-based inhibitors.

Gold-containing drugs, which have been used in medicine for ages, have been validated as potent and irreversible *in vitro* TrxR inhibitors in the nanomolar range. The first gold-containing compounds that were proven to affect TrxR were the gold(I)-thiolate drugs, utilized in the treatment of rheumatoid arthritis. The interaction of gold(I) with TrxR was first addressed by Hill and collaborators, which were the first authors reporting the effect of aurothioglucose (**1**, Figure 2) on rat TrxR [17]. Concomitantly, Gromer *et al.* studied aurothioglucose and auranofin (**2**, Figure 2) as inhibitors of purified human TrxR1 [18]. Rigobello *et al.* reported that auranofin is also proficient in inhibiting mitochondrial rat TrxR2, thus leading to stimulation of permeability transition and mitochondrial swelling in isolated purified mitochondria [19,20], as well as oxidative stress, cytochrome c release and cell death through apoptosis in human cancer cells [21,22].

Although gold(I) derivatives have been shown to exert both glutathione peroxidase (GPx) and TrxR inhibitory action by forming a three-coordinate intermediate gold(I)–selenolate complex [23,24,25,26], TrxR is far more susceptible toward inhibition by gold(I) compounds than the selenoenzyme GPx. Auranofin hampers TrxR activity in near stoichiometric concentrations, with a formal Ki of 4 nM [18], whereas GPx is inhibited in the micromolar range, thus requiring a 1000-fold higher concentration for its inhibition. The difference in enzyme inhibition has been related to the position of the Sec residue in these selenoenzymes, which is fairly more accessible in TrxR than in GPx. Hence, auranofin acts as a potent and more selective inhibitor of TrxR over GPx. In addition, gold complexes derived from the lead compound auranofin have demonstrated a considerable selectivity for the inhibition of TrxR over glutathione reductase (GR) or other structurally-similar enzymes. This selectivity is commonly attributed to the higher affinity of the gold center to selenium compared to sulfur, rendering the nucleophilic selenolate of reduced TrxR the prime target site of modification by this metal. This has also been experimentally confirmed by using mutant forms of TrxR, bearing a Cys residue in the place of Sec. These mutants were significantly less sensitive to inhibition by metallodrugs than the native proteins [27]. This enzyme selectivity exerted by auranofin against TrxR ideally fits with one of the most important paradigms in anticancer drug design, the activity towards a single macromolecular target that is overexpressed in cancer cells, thus making this drug a feasible candidate for cancer therapy. However, despite that fact, auranofin has only recently entered clinical trials as an anticancer agent for the treatment of recurrent epithelial ovarian, primary peritoneal or fallopian tube cancer [28]. The reason(s) that curtailed the use of auranofin in the treatment of cancer could be found in the severe clinical toxicity shown by this gold-based drug in arthritic patients, including proteinuria, diarrhea and bone marrow suppression [29]. In addition, the pharmacokinetic profile of auranofin appears to be significantly affected by the lability of the metal–thioglucose bond, which determines a weak stability of the complex into the blood and a rapid metabolization of the drug due to its conjugation to serum proteins, especially albumin [30,31]. On these bases, a more rational development of novel “auranofin-like” gold(I) complexes, encompassing the optimization of both phosphine and thiol ligands, has been pursued. Many highly promising novel gold(I) species have been reported, shedding also more light on the issue of structure-activity relationships (SAR’s). Keeping in mind that the lability of the thiolate group contributes to defining the biodistribution and kinetic properties of gold(I) complexes, we recently developed a series of linear, P–Au–X “auranofin-like” gold(I) Complexes **3**–**9** (Figure 2), maintaining the [Au(PEt_3_)]^+^ moiety and replacing the unstable thioglucose anion with other thiolates, as well as halogens (X) [32]. Ligands possessing a different binding strength to the gold center were employed, with the aim of investigating an eventual SAR effect based on the different stability of the Au–(X) bond. Although being more efficient against the cytosolic isoform than for the mitochondrial TrxR2, all of the tested compounds were able to selectively inhibit TrxR, with IC_50_ values in the low or sub-nanomolar range (IC_50_ values in the 0.31–1.8 nM range towards TrxR1 and in the 0.7–10 nM range towards TrxR2). Their efficacy in hampering TrxR in human ovarian cancer cells was correlated with the nature of the X ligand and its affinity to the Au(I) center. Actually, compounds with halogens, which are hard bases that can easily dissociate from the metal center and result in the formation of charged gold(I) species, showed difficulty in crossing the cellular membranes and inhibiting cancer cell TrxR. Conversely, the most potent compounds were those containing soft bases as X ligands, namely thiocyanate, cyanate, dithiocarbamate and xanthate ligands, showing a high ability to reach the intracellular compartment and to inhibit the selenoenzyme. On the other hand, these differences in cellular accumulation and enzyme hampering did not confer an extensive difference in cancer cell growth inhibition potential, as the IC_50_ values calculated for all derivatives were strictly comparable and in the low or sub-micromolar range. Only in the case of the cationic [Au(tu)(PEt_3_)][Cl] Complex **9**, a scarce cellular uptake and a significantly lower enzyme inhibition potency were greatly consistent with a cytotoxicity up to seven-times inferior compared to auranofin. This study pointed out that the potency of phosphine containing “auranofin-like” gold(I) complexes, of the type P–Au–X, should be tuned by modulating X ligand affinity to the Au(I) center, thus obtaining complexes that are endowed with different abilities to cross the cell membrane and reach the intracellular target.

Following the perspective of replacing the thiol-carbohydrate moiety with S-donor ligands displaying pharmacological activity themselves, Ott and coworkers developed a gold(I) phosphine complex containing a naphthalimide ligand, *N*-(*N*′,*N*′-dimethylaminoethyl)-4-mercapto-1,8-napthalimide (**10**, Figure 2) [33]. The choice of this ligand was driven by the presence of a heterocyclic naphthalimide core and a side chain containing a protonable nitrogen, which enable DNA intercalation and binding to the DNA phosphate backbone, respectively. Although the luminescent Compound **10** was a slightly weaker inhibitor of TrxR when compared with the parental compound Et_3_PAuCl (*in vitro* IC_50_ of 0.27 and 0.14 μM for **10** and Et_3_PAuCl, respectively), it was capable of accumulating strongly and more efficiently in cancer cell nuclei in comparison to Et_3_PAuCl, hence allowing a more potent cytotoxic effect against breast and colon cancer cells. Nevertheless, despite the fact that a 26-fold increase in cancer cell accumulation was achieved with **10** in MCF-7 cells, the cytotoxicity profile of **10** exceeded that of Et_3_PAuCl by only 2.5 times.

In an attempt to improve the solubility, stability and bioavailability of the previously reported chlorinated phosphole-containing gold(I) Complex **11** (Figure 2), Viry *et al.* reported on a phosphole-containing gold(I) complex bridging a diverse thiosugar, 1-thio-β-d-glucopyranose 2,3,4,6-tetraacetato-*S*({1-phenyl-2,5-di(2-pyridyl)phosphole gold) (**12**, Figure 2) [34]. Complex **12** was capable of selectively inhibiting TrxR in MCF-7 cells (IC_50_ value of 1.9 μM), showing a cytotoxicity potency in the low-micromolar range. Subsequently, the authors proved that **12** was effective at inhibiting a malignant glioma growth *in vivo*, in a C6 glioma rat model [35]. Interestingly, derivative **12** was significantly more effective than the corresponding chlorinated compound, **11**. On the other hand, besides being both TrxR inhibitors, the gold(I) chloro- and thiosugar-substituted phospholes have been shown to also interact *in vitro* with DNA, albeit the latter more weakly. Hence, the mechanism accounting for their cancer cell killing effect may rely on both TrxR inhibition and DNA interaction. Serebryanskaya and co-workers subsequently reported the evaluation of a series of tetrazole-containing triphenylphosphane and triphenylfurane gold(I) Complexes **13**–**17** (Figure 2) [36]. The introduction of a 1(*R*)-5-thiotetrazolate ligand led to the obtainment of effective and selective inhibitors of TrxR, with *in vitro* enzyme IC_50_ values in the low nanomolar range. The cytotoxicity of all derivatives were in the low micromolar level, with IC_50_ values against breast and colon cancer cells ranging from 8.5–13.3 μM.

Based on the fact that one of the main limitation of gold(I) compounds is their poor water solubility, which can hinder *in vivo* bioavailability, Dyson and co-workers synthesized a series of linear P–Au–S gold(I) complexes containing hydrophilic water-soluble phosphine ligands, namely 1,3,5-triaza-7-phosphaadamantane (PTA), 3,7-diacetyl-1,3,7-triaza-5-phosphabicyclo[3.3.1]nonane (DAPTA) and sodium triphenylphosphine trisulfonate (TPPTS) [37]. All Complexes **18**–**24** (Figure 2) elicited *in vitro* TrxR inhibitory effects (IC_50_) in the low nanomolar range, being more efficient against the cytosolic isoform (0.62–1.92 nM range) than for the mitochondrial TrxR2 (4.12–6.95 nM range). Notably, no large variations in the ability to inhibit TrxR were detected among all seven gold compounds, which all elicited IC_50_ values against TrxR1 very similar to those obtained with auranofin. All derivatives showed a cytotoxic activity in both cisplatin-sensitive and -resistant ovarian cancer cells, with IC_50_ values in the 4–16 µM range. However, their antiproliferative activity was inferior to that produced by auranofin, thus suggesting that the coordination of highly hydrophilic phosphine ligands to the Au(I) metal center does not confer a higher cytotoxic profile, at least *in vitro*. In addition, Ortego and collaborators developed a series of thiolate-aminophosphine gold(I) Complexes **25**–**31** (Figure 2) [38]. Their results clearly suggested that the characteristics of the aminophosphine group coordinated to the gold(I) atom strongly affects their ability to inhibit TrxR. Even if all complexes exhibited a high antiproliferative potential in the low micromolar range (0.34–18.96 μM), no clear relationships between cytotoxic activity and the TrxR inhibition extent were found. Actually, the most cytotoxic Complex **31** (IC_50_ of 0.34 μM against HeLa cells) inhibited the TrxR enzymatic activity to a lesser extent (38%) than Complexes **25** (77%) and **27** (84%), the latter eliciting IC_50_ values of 1.7 and 3.29 μM against HeLa cells, respectively. On the contrary, the introduction of a thiosemicarbazone ligand in place of the thiolate led to the preparation of gold(I) Complexes **32**–**35** endowed with a reduced ability to target TrxR, with *in vitro* enzyme IC_50_ values in the micromolar range (Figure 2) [39].

Later, a substitution of the thiolate moiety with P, N, O and C donor ligands was attempted by many authors, with the aim of obtaining compounds showing a suitable stability and solubility under physiological conditions. Galassi and co-workers developed a series of pyrazole and imidazole phosphane gold(I) Compounds **36**–**43** (Figure 2) containing either hydrophilic (PTA) or lipophilic (PPh_3_, triphenylphosphane) phosphane ligands [40]. These gold(I)–phosphane complexes inhibited both isolated cytosolic (IC_50_ values in the 3.5–86.54 nM range) and mitochondrial (IC_50_ values in the 33.57–258.30 nM range) TrxRs at concentrations that scarcely affected the GPx and GR. Again, TrxR1 was more susceptible to gold(I) derivatives than the corresponding mitochondria isoform. Interestingly, when compared with the PTA derivatives, the hydrophobic PPh_3_ analogs appeared to be much more cytotoxic against cancer cells, with mean IC_50_ values in the submicromolar range. Among the PPh3 series, by increasing the deactivation potency of the substituents on the pyrazole ring, an increase in the antiproliferative activity was achieved (**37** > **36** > **40**). This effect was less marked in terms of TrxR inhibition, the TrxR hampering ability of derivative **36** being slightly higher than that elicited by **37**. Based on evidence that bis-chelated gold(I) phosphine complexes have shown great potential as anticancer agents, Rackham and collaborators studied the TrxR inhibitory activity and the anticancer potential of a bis-chelated Au(I) bidentate phosphine complex of the novel water-soluble ligand 1,3-bis(di-2-pyridylphosphino)propane (d_2_pypp) (**44**, Figure 2) [41]. They showed that Compound **44** inhibited the activities of both isolated Trx and TrxR at a low micromolar level and that this effect was accounting for its preferential cytotoxicity against cancer cells, Trx and TrxR inhibition being more marked in breast cancer cells than in mammary epithelial cells. Subsequently, Wetzel and co-workers developed a series of linear and tetrahedral bis-chelated gold(I) complexes of the type [(diphos)(AuCl)_2_] and [(diphos)_2_Au]X, with imidazolyl- and thiazolyl-based water-soluble diphos-type ligands [42]. Although all [(diphos)_2_Au]X derivatives were able to selectivity target TrxR in *in vitro* screenings (IC_50_ in the 0.12–1.22 µM range), the bis-chelated gold(I)-complex possessing an intermediate lipophilicity (logD = 1.41) showed the highest *in vitro* antitumor activity, eliciting IC_50_ values in the sub-micromolar range against ovarian adenocarcinoma cells sensitive and resistant to cisplatin. Later, Wang and collaborators described a novel soluble bis-chelated gold(I)−diphosphine Compound **45** (Figure 2) with a strong and selective anticancer activity, with IC_50_ values in the low micromolar range against a broad spectrum of cancer cell lines [43]. Remarkably, derivative **45** was able to significantly reduce tumor growth in several tumor xenografts models, without inducing severe adverse effects. Studies on its mechanism of action confirmed that it specifically inhibits isolated TrxR at nanomolar levels, by binding to the C-terminal redox site, without targeting other well-known selenol- and thiol-containing biomolecules.

With the aim of gaining more insight into the biological effects obtained by modulating the coordination environment and the physicochemical properties of gold(I) complexes, Santini *et al.* prepared and characterized three water-soluble cationic gold(I) homoleptic phosphine complexes of the type [Au(L)_4_]^+^, where L is thp = tris(hydroxymethyl)phosphine, PTA, or thpp = tris(hydroxypropyl)phosphine [44]. Among the tested cationic compounds, the most hydrophilic thp derivative **46** showed the highest cytotoxic activity (average IC_50_ value of 19.68 µM) and was able to induce a 50% decrease of TrxR activity *in vitro* at 4.2 nM. Likewise, Lupidi and co-workers developed a mixed phosphine gold(I) Complex **47** (Figure 2) containing tris(tert-butyl) phosphine (tBu_3_P) and bis(diphenylphosphino)ethene (dppet) ligands [45]. The compound was endowed with a high *in vitro* antitumor efficacy against human colon cancer cells, and its ability to inhibit TrxR in human colon cancer cells at low micromolar levels in a dose-dependent manner was also established. Later, Meyer and co-workers focused their attention on a series of six alkynyl phosphine gold(I) Complexes **48**–**53** (Figure 2). All derivatives demonstrated a significant antiproliferative activity, with IC_50_ in the low and sub-micromolar range (0.8 and 12.0 µM), and were able, although to different extents, to selectivity inhibit TrxR *in vitro* at nanomolar concentrations (IC_50_ values in the 0.045–1.4 µM range) [46]. However, as outlined for other derivatives, the authors evidenced that the differences in activity against TrxR did not always translate to a substantially higher cytotoxic activity.

**Figure 3:**
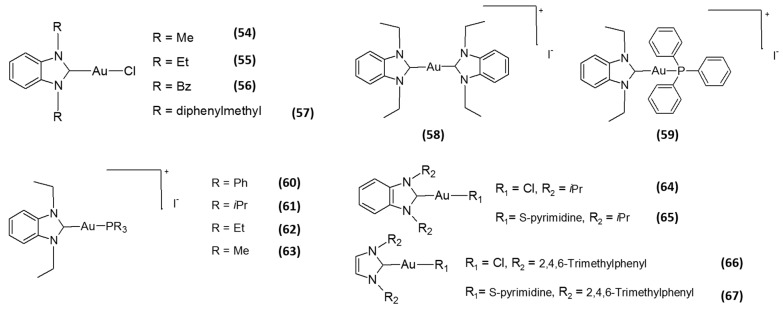
Structures of *N*-heterocyclic carbene (NHC) gold(I)-based inhibitors.

Motivated by the great potential of *N*-heterocyclic carbene (NHC) metal complexes in drug design and owing to NHC ligands’ similar donor properties to phosphines, very recently, several researchers have focused their attention towards the development Au(I) NHC as TrxR inhibitors. This class of derivatives exhibits very encouraging results, being able to efficiently and selectively inhibit TrxR. Among them, the linear cationic Au(I) NHC complexes, developed by the Berners-Price group, showed very interesting biological properties, including the selective inhibition of TrxR, a preferential cytotoxicity against tumor cells over normal ones and the triggering of apoptotic cell death [47]. Interestingly, the degree of selectivity against tumor cells correlated well with their log *P* values, with the compound possessing intermediate lipophilicity showing the most optimal selectivity and cytotoxic potency compared with the other two compounds. Following the success obtained by Berners-Price, Ott and collaborators developed a series of gold(I) complexes with benzimidazole-derived NHC ligands **54**–**57** (Figure 3) [48]. These mono-NHC gold(I) complexes were proven to selectively inhibit TrxR at low- and sub-micromolar concentrations (IC_50_ values in the 0.009–4.0 μM), even though to a lesser extent compared with gold(I) phosphine derivatives, *i.e.*, auranofin, triethylphosphine gold chloride (TEPAuCl) and TPPAuCl. On the other hand, these mono-NHC gold(I) complexes were shown to induce an antiproliferative effect at micromolar doses against different cancer cell lines, with IC_50_ values in the 4.6–60.7 μM range. Remarkably, they retained an adequate stability against thiols under biological conditions, a property that is highly desirable in the drug development of novel metal-based drugs. The same authors subsequently replaced the chloride ligands of the most cytotoxic derivative **55**, introducing ligands endowed with a higher coordinative stability, namely an additional NHC or a PPh_3_ ligand [49]. The coordination with these neutral ligands led to the formation of cationic species (**58** and **59**, Figure 3), retaining the ability to preferentially inhibit TrxR over structurally-related enzymes. The neutral chlorido derivative exhibited, beside a stronger and selective TrxR inhibition (*in vitro* IC_50_ of 0.36 µM) compared to the cationic Au(I) NHCs, an intensive binding to albumin, similar to that of auranofin. Conversely, the cationic complex **58**, with two NHC ligands, elicited a weaker inhibition of TrxR (*in vitro* IC_50_ of 4.89 µM), but retained a very scarce ability to bind albumin. Finally, the PPh_3_ derivative **59** led to a stronger inhibition of TrxR (*in vitro* IC_50_ of 0.66 µM) and an elevated protein binding ability. Accordingly, the mitochondrial inactivation (determined as the inhibition of mitochondrial respiration and mitochondrial membrane depolarization) against cancer cells was dependent on the type of coordinated ligand and the charge of the complexes. Concerning cancer cell selectivity, only Compound **58** showed a certain preference for tumor cells over non-transformed ones. This study thereby suggests that the introduction of ligands endowed with a different coordinative stability and/or the formal charge of the complexes could produce significant changes in the enzyme interaction profile of gold(I) NHC complexes, as well as on their putative pharmacokinetic behavior. In this respect, some of these authors later reported that the introduction of different alkyl residues at the phosphorus atom led to major changes in the biological activity and cellular bioavailability [50]. In particular, the bioactivities of Compounds **60**–**63** (Figure 3) were correlated with both complex reactivity and the extent of cellular accumulation, the latter being dependent to the lipophilicity of the substituents. Concerning TrxR inhibition, all derivatives were able to hamper 50% of the TrxR activity at sub-micromolar concentrations (0.03–0.66 µM), and by decreasing the size of the residues at the phosphorus atom, a stronger inhibition of TrxR was achieved. On the contrary, the most cytotoxic compound of the series was **60**, bearing the triphenylphosphane moiety, with IC_50_ values in the sub-micromolar range. This discrepancy was tentatively explained by the authors taking into consideration that, besides TrxR, PARP-1 was identified as an additional molecular target accounting for the bioactivity of these complexes. Subsequently, the same authors also demonstrated that the coordination of a naphthalimide moiety, which they had previously shown to confer DNA binding and nuclei accumulation abilities to the gold(I) complexes [33], in the imidazole-based NHC fragments of a chloride gold(I) derivative, led to the obtainment of metallodrugs with TrxR inhibitory activity in the submicromolar range and endowed with antiproliferative potential [51]. On the other hand, studies on a family of gold(I) NHC complexes containing 1,3-substituted imidazole-2-ylidene or benzimidazole-2-ylidene and chloro or 2-pyrimidinethiolato ligands revealed that halogen derivatives **64** and **66** (Figure 3) were less effective in inhibiting TrxR (IC_50_ values against isolated TrxR1 of 0.7 and 0.205 nM, respectively) compared to the corresponding thiolato complexes **65** and **67** (Figure 3) (IC_50_ values against isolated TrxR1 of 0.062 and 0.0184 nM, respectively), and this difference in hampering selenoenzyme activity was consistent with a reduced cytotoxic potential [52]. Overall, the majority of the above-listed gold(I) complexes have been proven to inhibit TrxR1 much more effectively than TrxR2 *in vitro*. However, some cationic gold(I) compounds, owing to their ability to accumulate inside mitochondria as a result of the high mitochondrial membrane potential (Δψm), have been proven to specifically inhibit TrxR2 in cells [53].

**Figure 4:**
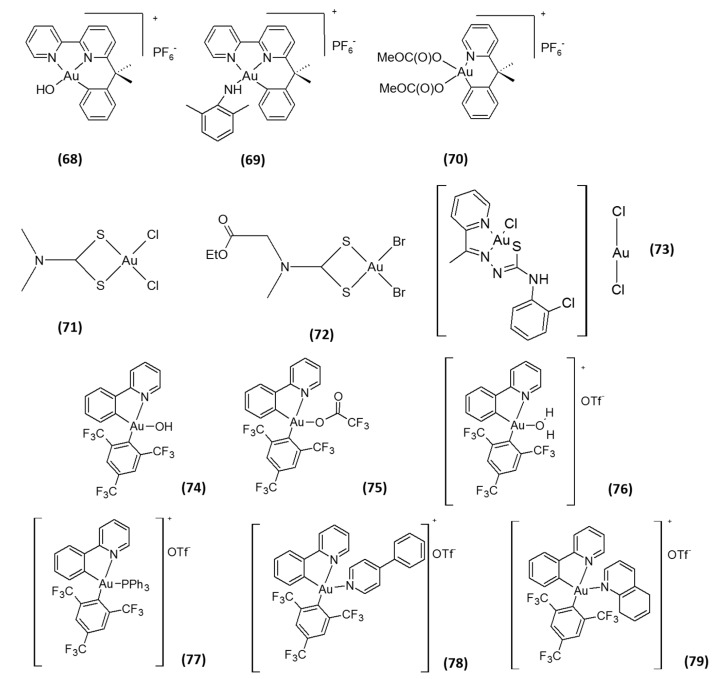
Structures of gold(III)-based inhibitors.

In addition to gold(I) complexes, gold(III) species have also attracted attention as TrxR inhibitors. Compounds belonging to this class exhibit a high *in vitro* anticancer effect due to TrxR inhibition, the latter occurring either by oxidation of key enzyme residues or by gold coordination to the C-terminal active site [26]. Among them, organogold(III) complexes with diamine ligands AuBiPy, AuXil and AuPy (**68**–**70**, Figure 4) were demonstrated to be inhibitors of isolated TrxR2 at low- or sub-micromolar doses (IC_50_ values of 0.28, 0.21 and 1.42 µM, respectively), and this inhibition was related to their cytotoxicity towards human cancer cells [54,55]. Engman and co-authors, described some organogold(III) complexes as very effective in inhibiting TrxR *in vitro* (IC_50_ values in the nanomolar range), but lacking any significant anticancer activity in cells and in *in vivo* xenograft models [56]. On the contrary, gold(III) dithiocarbamato complexes **71** and **72** (Figure 4) were shown to inhibit TrxR in prostate cancer cells at low micromolar doses and possess a high antitumor activity, both *in vitro* and *in vivo* [57]. Furthermore, the proteasome has been identified as a major *in vitro* and *in vivo* target of these complexes, thus suggesting that the antitumor efficacy possibly can be mediated by multitargeted mechanisms. Similarly, a gold(III) complex with 2-acetylpyridine-*N*(4)-ortho-chlorophenyl-thiosemicarbazone (**73**, Figure 4) was proven to be very effective in hampering TrxR activity (*in vitro* IC_50_ of 0.23 µM) [58]. By comparing the TrxR inhibition results with those of the cytotoxicity studies, however, a correlation could not be established, and the cytotoxic profile of the compound seems to mainly be the result of the coordination of thiosemicarbazone ligand. Recently, a series of phosphorescent (2-phenyl)pyridine gold(III) 2,4,6-tris(trifluoromethyl) phenyl Complexes **74**–**79** (Figure 4) were investigated by Rubbiani and co-authors [59]. These complexes showed a moderate TrxR inhibition, with IC_50_ values in the micromolar range (1.02–22.9 µM). Interestingly, most of the Au(III) compounds displayed a significantly lower activity towards GR. Nevertheless, no direct correlation could be drawn looking at cytotoxicity and TrxR inhibition activities, the most effective TrxR inhibitor Compound **76** (*in vitro* IC_50_ of 1.02 µM) being less cytotoxic compared to the weaker TrxR inhibitor **75** (*in vitro* IC_50_ of 1.43 µM). In addition, a DNA binding ability was suggested to contribute to their cellular effects. Overall, the studies performed so far on gold(III) complexes underlined that this class of derivatives acts through a multitargeted mechanism that involves interactions with molecular targets other than TrxR inhibition [60,61,62].

## 3. Platinum-Based Inhibitors

**Figure 5:**
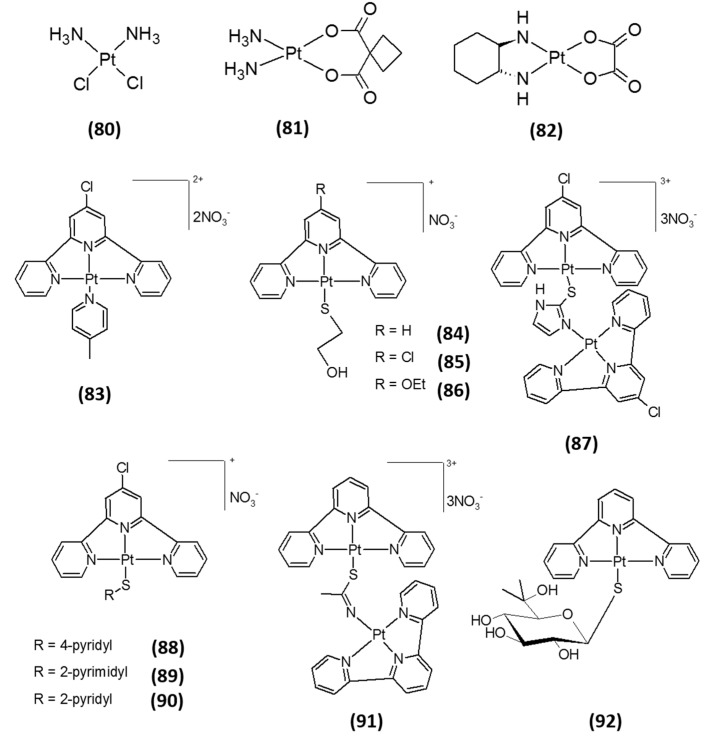
Structures of platinum(II)-based inhibitors.

The cytotoxic effect of cisplatin (*cis*-diamminedichloroplatinum(II) (CDDP), **80**; Figure 5), the milestone of metal-based drugs in cancer chemotherapy, is generally considered to be attributed to the formation of DNA-platinum adducts, causing cell cycle arrest and triggering of apoptosis [63]. However, since only a very small fraction of the intracellular CDDP has been found to react with genomic DNA, other biological targets have been proposed to take part, accounting for the cytotoxic effects observed [64]. Since the active metabolites of cisplatin are strong electrophiles, they can also react with nucleophiles other than DNA, such as RNA or nucleophilic moieties of proteins. Of specific interest are the numerous pieces of evidence showing that interactions with the intracellular disulfide/dithiol systems may account for some of the cytotoxic effects exerted by cisplatin. In this respect, the Sec residue of selenoproteins may represent a major target [65]. In accordance, it has been shown that an increase in Trx system activity contributes to cancer cell resistance to CDDP [10,22], and that at concentrations ranging in the micromolar range, CDDP possesses TrxR inhibitory activity [66]. Interestingly, CDDP has no effect on oxidized TrxR, *E. coli* TrxR, engineered Sec498Cys mutant TrxR, nor on GR, thus suggesting that its mechanism of inhibition most likely involves coordination of platinum to the Sec-containing redox center [65,67,68]. In addition, carboplatin (*cis*-diammine(1,1-cyclobutanedicarboxylato)platinum(II), **81**, Figure 5) and oxaliplatin ([(1*R*,2*R*)-cyclohexane-1,2-diamine](ethanedioato-*O*,*O*′)platinum(II), **82**, Figure 5) were also found to be effective inhibitors of isolated TrxR at the micromolar level [68]. Yet again, both Pt(II) derivatives exerted a superior inhibition of TrxR over the related flavoprotein enzyme GR.

In recent years, Becker *et al.* evaluated a series of platinum(II) terpyridine Complexes **83**–**92** (Figure 5), known for their well-documented DNA-intercalating activity [69], as TrxR inhibitors. Notably, all complexes were capable of accomplishing an irreversible TrxR inhibition in a dose-dependent manner, eliciting low nanomolar IC_50_ values in a cell-free system and low micromolar concentrations in cancer cells [70]. Similarly, other authors designed and developed novel platinum(II) terpyridine and nitrofuran complexes able to hamper the activity of isolated human TrxR1 in the nM range [27,71,72]. All together, these studies suggest that the cytotoxic properties of these classes of derivatives are likely to result from both DNA interaction and other mechanisms of action, possibly involving a specific and irreversible TrxR inhibition.

## 4. Silver-Based Inhibitors

**Figure 6:**
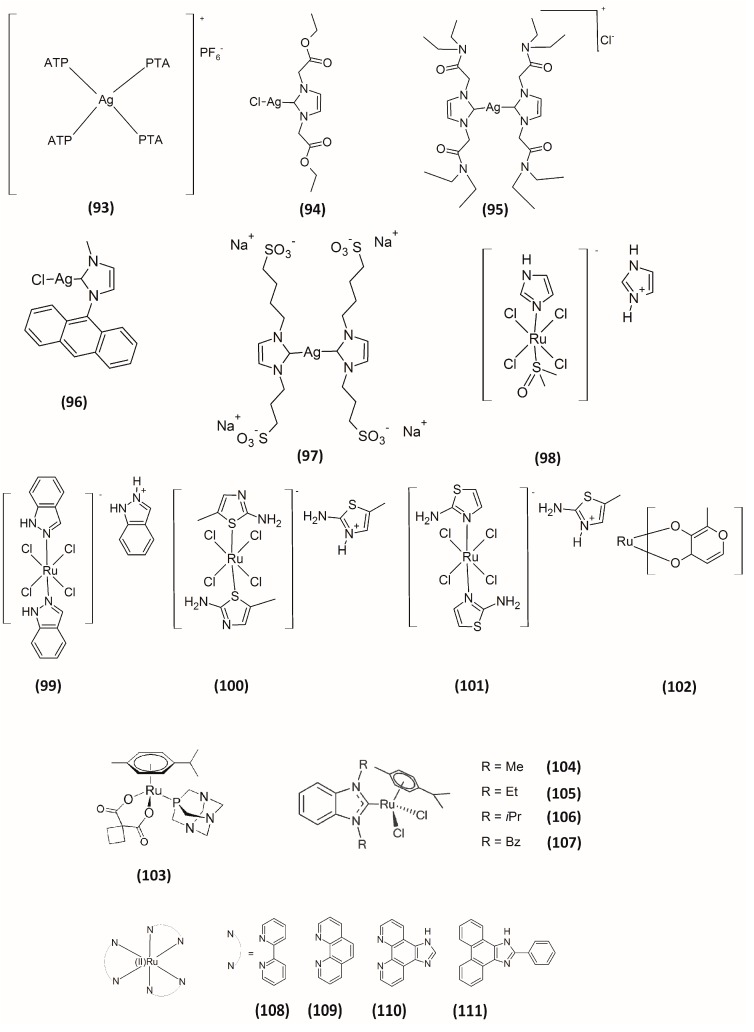
Structures of silver-based and ruthenium-based inhibitors.

Even though silver has been used as colloidal material for many years, only recent advances in synthesis and characterization techniques have led to this metal being incorporated into many metal complexes for drug development purposes. Silver complexes, which have been widely studied as anti-infectives in the last decade, have recently been proposed as potential anticancer agents [73]. Given the knowledge that exposure to Ag leads to the accumulation of Ag_2_Se in mammals [74] and keeping in mind the reactivity of Sec residues toward electrophilic compounds, we recently evaluated some silver(I) complexes as TrxR inhibitors **93**–**95** (Figure 6) and compared their activity with those elicited by their gold(I) parent compounds [44,75]. Like their corresponding gold(I) complexes, the silver(I) derivatives were effective in inhibiting 50% of isolated TrxR1 activity at nanomolar concentrations. Remarkably, the cytotoxic effects induced by Ag(I) complexes were slightly higher than those elicited by corresponding Au(I) complexes. Furthermore, the silver(I)-NHC derivative **94** showed a preferential cytotoxic activity *vs.* neoplastic cells. Similar results were later obtained by Rigobello and co-workers by comparing some silver(I)- and gold(I)-NHC complexes bearing a fluorescent anthracenyl ligand (**96**, Figure 6) [76]. In this report, the dimerization of peroxiredoxin 3 was also observed, thus demonstrating the ability of the silver compound to reach the mitochondrial compartment. Subsequently, we reported on a water-soluble sulfonate-functionalized silver(I) NHC **97** with a significant *in vitro* antiproliferative activity (in the sub-micromolar range) that was correlated with its strong ability to inhibit TrxR [77]. The inhibition of this selenoenzyme induced by Compound **97** (Figure 6) consistently determined an alteration of the cellular redox environment, thus leading to the induction of apoptotic cell death through the activation of the ASK-1 pathway. Even though, at present, mechanistic aspects concerning the mode of inhibition are still lacking, taken together, these studies validate the hypothesis of TrxR as a protein target for silver(I) in addition to gold(I) complexes.

## 5. Ruthenium-Based Inhibitors

Ruthenium-based compounds have emerged in recent years as potential candidates for cancer treatment. Currently, the Ru(III) complex NAMI-A, [H_2_im][*trans*-Ru(III)Cl_4_-(DMSO-S)(Him)] (Him = imidazole, **98**; Figure 6) and KP1019 ([H_2_ind][trans-Ru(III)Cl_4_(Hind)_2_] (Hind = indazole, **99**; Figure 6) have recently entered clinical trials [78,79]. Concerning their mechanism of action, many studies have demonstrated that Ru complexes exert their antiproliferative activities primarily through interaction with DNA and with different cellular proteins [80]. Among them, TrxR has also been proposed as a molecular target for this class of complexes. Actually, Ru complexes could inhibit TrxR activity due to the “soft” character of the Ru center. Messori and co-workers originally reported on a series of ruthenium(III) compounds able to inhibit TrxR. Based on the structure of NAMI-A, they synthesized some “tetrachlororuthenate” Ru(III) Compounds **100**–**102** (Figure 6) [81]. However, compared to gold- or platinum-containing complexes, these ruthenium(III) complexes were significantly less potent in inhibiting TrxR1, showing IC_50_ values in the micromolar range, and were not effective against mitochondrial TrxR2 isoform. A year later, the same authors reported a series of ruthenium(II)–arene compounds as inhibitors of TrxR [82]. Among them, the most efficacious derivative **103** (Figure 6) elicited an inhibition of 50% TrxR activity at low micromolar concentrations, with IC_50_ values of 4.6 μM and 14.7 μM for TrxR1 and TrxR2, respectively. In the search of some preliminary SARs, the authors pointed out that the higher the steric hindrance effect of the arene moiety, the lower the observed inhibitory potency of TrxR was, indicating that bulky substituents in the arene ligand interfered with the effective interaction between the compounds and the enzyme. Nevertheless, the authors concluded that these derivatives behave much more as inhibitors of cathepsin B rather than TrxR inhibitors. Likewise, Ott and co-workers described some arene Ru(II) complexes **104**–**107** (Figure 6) endowed with TrxR and cathepsin B inhibitory activity at micromolar range [83]. These authors indicated a preference of their compounds for the inhibition of the selenoenzyme TrxR compared to the cysteine-rich cathepsin B, with the IC_50_ calculated for TrxR being up to 50-times lower compared with those calculated for cathepsin B. Accordingly, the cytotoxic effects on tumor cell growth were accompanied by an appropriate decrease in cell impedance and cellular respiration. The studied complexes were, however, also able to efficiently bind to BSA and DNA, thus pointing out that their *in vitro* antiproliferative activity might be based on a multitargeted mechanism. Later, Luo and co-workers prepared a series of Ru(II) polypyridyl complexes with diimine ligands **108**–**111** (Figure 6), and their interaction with TrxR was examined [84]. Despite these complexes showing the ability to inhibit TrxR at micromolar concentrations, their cytotoxic potency were significantly lower compared to that of cisplatin.

## 6. Other Metal-Containing Inhibitors

**Figure 7:**
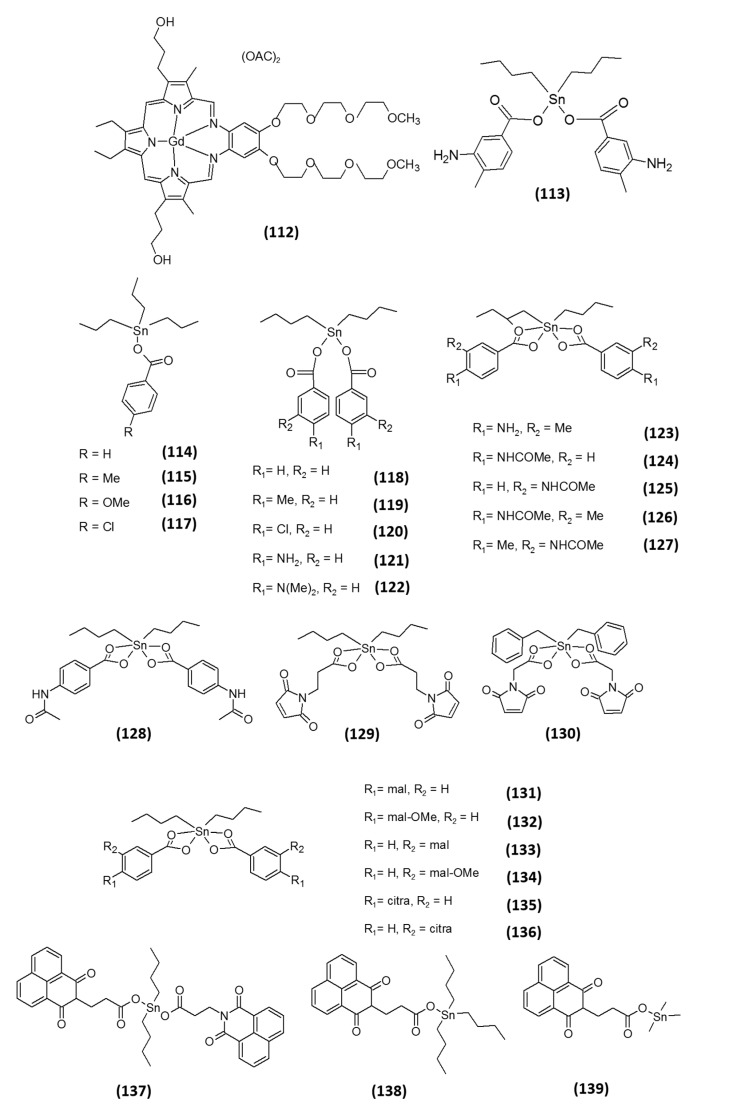
Structures of other metal-based inhibitors.

Motexafin gadolinium (MGd, **112**; Figure 7) is a porphyrin-like molecule utilized in cancer therapies [85]. MGd is currently in clinical trials, both as a single drug and in combination with other chemotherapeutics or with radiotherapy for the treatment of different types of cancers, including non-small cell lung, brain, renal, pancreatic and biliary carcinomas and lymphoma. The anticancer activity of this compound has been correlated with its ability to undergo redox cycling and to generate superoxide and other ROS under aerobic conditions causing oxidative stress. MGd is also able to catalyze the oxidation of intracellular reducing metabolites, such as NADPH, GSH, ascorbic acid and protein vicinal thiols [85]. It has further been shown to induce cytotoxicity in multiple myeloma cell lines by altering the cellular redox state due to an increased production of ROS [86]. In 2006, the effects of MGd on cancer cells’ redox state was associated with its ability to interact with TrxR [87]. Holmgren and co-workers showed that MGd is capable of interacting with TrxR1 and of generating ROS, such as superoxide and hydrogen peroxide. In addition, however, in the same study, the authors demonstrated that it also acts as a potent inhibitor of ribonucleotide reductase, a cellular enzyme essential for DNA synthesis.

Recently, the group of Prof. Ott, through a virtual screening study for active TrxR inhibitors, selected the tin(IV) organometallic compound NIH 643845 (**113**, Figure 7) as one of the most active compounds. Based on the virtual screening findings, they thought it of interest to provide a “proof of concept” for the involvement of TrxR inhibition in the antitumor activity of tin(IV) species [88]. Accordingly, they studied the effects on TrxR of a series of benzoate tin organometallics related to Compound **113** and laid down some preliminary, but fundamental, SARs. All *n*-butyltin(IV) complexes **114**–**127** (Figure 7) inhibited TrxR with IC_50_ values in the micromolar range, the most active derivatives eliciting IC_50_ values in the low micromolar range (1–5 µM). It is important to mention that TrxR inhibitory activity was partially modulated by variation of the substituents on the benzoate ligands. Among all derivatives, tri-*n*-butyltin carboxylates (**114**–**117**) were on average weaker inhibitors (*in vitro* IC_50_ values in the 5.88–67.16 µM range) than the respective di-*n*-butyl derivatives (**118**–**127**) (*in vitro* IC_50_ values in the 1.37–17.59 µM range), and among the latter, *N*-acetylated derivatives **124**–**127** (*in vitro* IC_50_ values in the 1.37–4.70 µM range) were more active than complexes with a free or methylated amino group, **121**–**123** (*in vitro* IC_50_ values in the 10.41–16.05 µM range). The authors attributed these differences to the presence of an amide bond in the *N*-acetylated derivatives, which can decrease the electron density of the carboxylate groups, thus destabilizing their coordination to tin and ultimately resulting in stronger inhibition of TrxR. On the other hand, the position of the substituents on the aromatic rings was shown not to influence the TrxR inhibitory activity. As reference compounds, they used the di-*n*-butyltin(IV) oxide and the tri-*n-*butyltin(IV) chloride. The former was proven completely ineffective, whereas the latter exhibited similar activity to the carboxylates. On these bases, the authors suggested the presence of an appropriate leaving group (a chloride or a carboxylate) as an essential requirement for obtaining an effective tin(IV)-based TrxR inhibitor. However, substantial differences in TrxR hampering ability did not always confer a huge difference in the cell growth inhibitory effects against cancer cell lines, the less efficient TrxR inhibitor being derivative **117** (Figure 7) and the more cytotoxic than the most effective at hampering TrxR being derivative **124** (Figure 7) (TrxR IC_50_ values of 67.16 and 1.37 µM, respectively; IC_50_ against MCF-7 cells of 0.13 and 0.60 µM, respectively).

Subsequently, the same authors prepared a series of organotin(IV) carboxylate complexes **128**–**139** (Figure 7) with naphthalimide-, citraconimide- or maleimide-derived ligands. As TrxR inhibitors, three of the four newly prepared maleimide derivatives were more effective compared with both citraconimide- and naphthalimide-containing compounds, eliciting IC_50_ values in the 2.3–11.3 µM range [89]. Even though all of these compounds showed a TrxR inhibitory activity at µM concentrations and some SARs were tentatively drawn, again, no clear correlation was established between TrxR inhibition and cytotoxicity potency, thus indicating that inhibition of TrxR is unlikely the main mode of action of these compounds.

## 7. Semimetal-Based Inhibitors

The use in medicinal chemistry of tellurium, in the form of 2 KTeO_3_^2−^, started during the 1930s with the work of Sir Alexander Fleming, who reported that it was an effective antibiotic able to act even in penicillin-resistant bacteria [90]. However, its potential as an anticancer agent is relatively unexplored.

Engman and co-workers synthesized several organotellurium compounds, where Te is in the +IV oxidation state, including water-soluble organotellurium compounds (diaryl tellurides, alkyl aryl tellurides and dialkyl tellurides), tellurium analogues of vitamin E, organotellurium steroids, lipids, amino acids, nucleic bases and polyamine inhibitors, as well as cyclodextrin-derived diorganyl tellurides [91,92,93,94], and studied their interaction with TrxR. Notably, some of these organotellurium derivatives inhibited the isolated enzyme at sub- or low micromolar levels. However, their TrxR inhibition did not correlate well with their cytotoxicity.

Recently, the group of Prof. Holmgren has demonstrated that arsenic trioxide (ATO) is a potent inhibitor of TrxR [95]. ATO has been used for several centuries in traditional medicine for curing cancers, and recently, it has been shown to be very effective in the treatment of both leukemia [96,97,98,99], and solid tumors [100]. In fact, in 2010, the FDA approved it for the treatment of acute promyelocytic leukemia. Past evidence indicated protein sulfhydryl groups as the main targets of the drug [100]. On the other hand, a mechanism involving a strong and irreversible inhibition of mammalian TrxR, involving both the C-terminal and N-terminal redox active sites of the enzyme, was recognized by Holmgren and co-workers. Notably, the inhibition of TrxR in breast cancer cells subsequently resulted in the inactivation of the Trx system and, ultimately, the induction of cell death.

## 8. Concluding Remarks

Since the discovery of TrxR inhibition by auranofin, great interest in the metal-containing compounds as TrxR inhibitors has emerged. Among all, gold(I) complexes are the most effective inhibitors of mammalian TrxRs known today. However, in recent years, many highly promising novel metal-based species have been described, which we herein have tried to briefly summarize as an attempt to give a general overview of the current development in the field. Several of these metal- and semimetal-based compounds have been shown to inhibit TrxR, and the anticancer potential of such compounds continues to be the subject of considerable research. As already mentioned, TrxR is strongly associated with tumor proliferation and tumor aggressiveness. It is further known to be upregulated in many tumors and linked to drug resistance. It is therefore not surprising that TrxR is believed to be a promising target for cancer therapy, and therefore, more extensive investigations are needed.

Clinically-approved platinum drugs have also been proven to exert their antitumor activity, not only through the interaction with DNA, but also by TrxR inhibition. Among metal-based derivatives, silver(I) complexes have emerged as very effective in TrxR inhibition. Owing to their selectivity towards cancer cells, they represent a very promising class of anticancer agents and deserve to be further examined. Actually, mechanistic studies proving Ag fragment binding to TrxR and the discovery of a silver(I) drug target site of TrxR are still lacking and are indeed warranted. Similarly, tin(IV) organometallic compounds show extraordinary anticancer properties, being able to selectively target TrxR. More *in vitro* and *in vivo* studies are nonetheless needed in order to fully elucidate the potential of these new classes of TrxR inhibitors for the development of new chemotherapeutic drugs.

Overall, however, some general considerations have to be underlined concerning metal complexes as TrxR inhibitors. The metal center has emerged as fundamental to achieve the inhibition of the TrxR enzymes, thus supporting the assumption of the preferential binding to the Sec residue present in the active site of TrxR. Nevertheless, it should be mentioned that a direct correlation between TrxR inhibition and cytotoxic effects of metal complexes could not always be claimed, and this indicates that other mechanisms besides TrxR inhibition may contribute to the overall pharmacological profile. In particular, structural modifications of metal complexes through the modulation of ligand physicochemical characteristics, as well as of the stability/lability of the coordinative bonds lead to major changes in the biological activity, pharmacodynamic properties and cellular bioavailability. Hence, through an accurate choice of the ligands, a fine tuning of the pharmacological profile can be accomplished. Based on these considerations, however, a key issue in the development of metal complexes as TrxR inhibitors is the evaluation of enzyme inhibition also in intact cells, since cell-free studies can be misleading and do not fully reflect their pharmacological potential.

Almost totally unexplored is, at present, the potential of semimetal compounds as anticancer agents targeting TrxR. The few reports on organotellurium derivatives, despite being very promising, are lacking both mechanistic and SAR aspects, thus pointing out that further studies are required to gain more insight into the development of semimetal-based derivatives as TrxR inhibitors. In addition, as several different splice variants and isoforms of both TrxR1 and TrxR2 have been characterized, more research effort is required to understand their specific role in tumor development and resistance. Increased knowledge will undoubtedly enable more accurate and effective drug development.
